# A Retrospective Review of Wild and Zoo-Housed Platypus Medical Records (1991–2024)

**DOI:** 10.3390/ani16060875

**Published:** 2026-03-11

**Authors:** Jessica Whinfield, Rebecca Vaughan-Higgins, Larry Vogelnest, Kristin Warren, Cheryl Sangster

**Affiliations:** 1 School of Veterinary Medicine, College of Environmental and Life Sciences, Murdoch University, Murdoch, WA 6150, Australia; 2 Centre for Terrestrial Ecosystem Science and Sustainability, Harry Butler Institute, Murdoch University, Murdoch, WA 6150, Australia; 3 Taronga Institute of Science and Learning, Taronga Conservation Society Australia, Mosman, NSW 2088, Australiacsangster@zoo.nsw.gov.au (C.S.)

**Keywords:** platypus, retrospective review, medical records, pathology, veterinary medicine, Australian wildlife

## Abstract

Understanding platypus health and disease is made challenging by the cryptic nature of this unique and iconic species. The retrospective medical record review methodology offers a valuable tool to better understand platypus health. A multi-institution review was performed, with 21 organisations and individuals contributing veterinary and pathology records spanning 34 years and 5 Australian states and territories. In total, records were reviewed from 278 wild platypuses and 40 zoo-housed platypuses, with a combined total of 383 presentations. Data from these were extracted and analysed, providing information on demography (age, sex), geographic location, season, reason for presentation, outcome of presentation, and clinical and pathological findings. For wild platypuses, key findings included that the juvenile age class was disproportionately represented in Queensland and New South Wales, and that the peak in juvenile presentations corresponded with weaning. For both wild and zoo-housed platypuses, novel reports of neoplasia were identified, and in wild platypuses, the first reports of neural angiostrongyliasis. For zoo-housed platypuses, an area identified for future research is the high prevalence of presentations for skin lesions. This study contributes to our understanding of platypus health and disease and should be used to guide further work to improve both conservation and welfare outcomes for one of the world’s most unique mammals.

## Simple Summary

Platypuses are unique and iconic Australian mammals. Because they are frequently nocturnal and spend significant time either in burrows or in water, it can be challenging to understand platypus health and diseases. We accessed and reviewed the veterinary records of 278 individual wild platypuses and 40 zoo-housed platypuses from 21 organisations and individuals, spanning 34 years. From these records, we looked at information including: age and sex; location and date of presentations; why the platypuses were presented to a veterinarian; whether they died or survived; and health and disease findings. Wild juvenile platypuses were more likely to be presented to veterinarians in the north of their range than in the south, with many juvenile presentations around the time of weaning. Skin conditions were common in zoo-housed platypuses. The records included diagnoses not previously reported in platypuses, including cancers and rat lungworm infection. This study increases our understanding of platypus health and disease, and we believe its insights will both support the species’ conservation and improve the welfare of zoo-housed platypuses.

## 1. Introduction

Platypuses (*Ornithorhynchus anatinus*) are a unique and iconic species, inhabiting freshwater ecosystems of eastern Australia, including the island state of Tasmania [1]. Platypuses are listed as near threatened by the IUCN, with a decreasing population trend [2]. This is reflective of their vulnerability to anthropogenic pressures, including changing land use (especially agriculturalisation and urbanisation), waterway regulation (including the construction of dams and weirs), entanglement and entrapment in rubbish and fishing gear, predation by introduced species (particularly dogs and foxes), and climate change-exacerbated natural disasters (such as drought, flooding, and fire) [1,2]. Platypuses are considered near extinct in South Australia, the western-most extent of the species’ distribution [1].

Platypuses are a cryptic species and inherently challenging to study, being semi-nocturnal, semi-aquatic, and semi-fossorial [1]. They are seasonal breeders, with the timing of reproduction earlier in the north of their range and later in the south. In New South Wales (NSW; approximately midway in their latitudinal distribution), courtship begins around August, followed by the female building the nest, and laying and incubating eggs. She then nurses the nestlings from hatching until their emergence from the nest at about four months of age, between late January and early March. In Tasmania (the most southerly extent of the platypuses’ range), emergence occurs two to three months later (late March to early May, i.e., autumn) [1,3,4]. At the time of writing, platypuses are held in zoos or sanctuaries at low numbers by nine institutions in Australia, and one in the United States of America [3]. To date, less than ten zoo-housed females have reproduced successfully.

Understanding platypus health and disease is important for: establishing a baseline of health risks; reducing risks associated with conservation actions such as translocations [5]; and improving welfare and outcomes for both rescued platypuses and those held in zoos or sanctuaries. However, understanding platypus health and disease is challenging due to difficulties in observing and sampling the species. This is further compounded by the relatively low numbers of individuals held in zoos or sanctuaries, and the significant limitations in extrapolating from similar species as a result of their unique biology; the closest living relative to platypuses are the echidnas (Family: Tachyglossidae), which are markedly different in their morphology, physiology, and ecology [6]. To date, peer-reviewed literature on platypus health and disease (excluding physiology studies) have included the following forms: case reports [7]; limited mortality case series (*n* = 20, [8]; *n* = 25, [9]); targeted surveillance for one or more pathogens or hazards in wild populations, coupled with health metrics [10,11,12,13]; and a limited number of retrospective reviews [14,15,16]. These studies have made significant advances in understanding platypus pathology; however, knowledge relating to platypus health remains poor when compared to other iconic Australian species, such as koalas [17], and rudimentary compared to domesticated species.

Retrospective medical record reviews are descriptive, observational studies that utilise data originally collected for purposes other than research, such as health care records [18,19]. The benefits of retrospective reviews include their low cost, lower resource requirements compared to other methodologies (for example, cohort studies), and the ability to study rare diseases or exposures, or uncommonly encountered species. This methodology is therefore popular in studies of human and animal health, including for both zoo-housed and free-ranging wildlife [18,19,20,21,22].

To date, there have been no systematic reviews of the medical records of zoo-housed platypuses, with the three previous retrospective reviews focusing on wild platypus morbidity and mortality records [14,15,16]. These three reviews have overlapping data sets and a limited geographic scope, focusing on Victoria either exclusively [14,15] or predominantly (78.0% of cases reviewed (305/391) in [16]). Only one of these reviews [14] included a veterinarian as an author, and none had a pathologist involved in authorship: the authors of [22] consider the involvement of veterinarians and pathologists essential for wildlife morbidity and mortality studies. [14] reviewed the veterinary medical records of 38 wild platypuses presenting to Healesville Sanctuary’s Australian Wildlife Health Centre (2000–2014). In comparison, the methodology used by [15,16] relied on “ad hoc” reporting of platypus mortalities from a range of sources (including members of the public, natural resource managers, biologists, and veterinarians), with or without supporting information (such as photos or the provision of carcasses). The latter study [16] included 23 mortality records from platypuses that presented to Healesville Sanctuary between 2005 and 2024, thus suggesting overlap with the records used in [14], although this study is not referenced. Despite the statement in [16] that they “develop(ed) a consolidated set of records to characterise the entire range of factors known to have contributed directly to platypus mortality in recent decades”, it is probable that there are additional morbidity and mortality causes beyond those reported in these three studies. Specifically, there is a risk of underestimating both the impact and diversity of disease on platypuses: between the three review studies performed to date, disease was only identified in three platypuses.

The aim of this project was to use the retrospective review methodology to conduct a distribution-wide study exploring morbidity and mortality trends for both zoo-housed and wild platypuses. This included trends relating to demography (age, sex), geographic location, season, reason for presentation, outcome of presentation, and clinical and pathological findings.

## 2. Materials and Methods

A retrospective medical record review was performed. The review had three main steps: record acquisition; record review and data extraction; and data analysis. The STROBE guidelines [23] are a checklist of recommendations designed to guide the reporting of observational research, and these were incorporated into the methodology where possible. The guidelines produced by [22] for performing retrospective morbidity and/or mortality studies in zoo-housed wildlife were also incorporated where possible.

### 2.1. Record Acquisition

The individuals and institutions considered most likely to hold platypus medical records were identified as those meeting one or more of the criteria described in Table 1 and were contacted by email. The platypus’s range states and territories were considered to be Queensland, New South Wales (NSW), the Australian Capital Territory (ACT), Victoria, Tasmania, and South Australia [1].

Organisations and individuals were initially contacted between April 2021 and January 2023. This extended period reflected additional points of contact being identified through both the record review process and through recommendations made by record holders. Individuals or institutions with relevant records who consented to participate shared records with the study’s first author in the format of electronic files or original paper documents. In some instances, travel to the institution by the first author was required to manually locate and copy paper records. Records were received for review between August 2021 and September 2024. This prolonged timeframe of record receipt was a product of multiple factors, including: the lag time between the request for records being made and the records being provided for review; individuals and institutions being contacted at different times; and some institutions continuing to submit additional records as cases presented, with these continuing to be included in the data set.

During the record acquisition period, a NSW-wide callout (through social media and by directly emailing key contacts) asked people to notify the first author of any dead wild platypuses found. Where possible, any platypuses that were found deceased were transported to Taronga Zoo, Sydney, where a gross necropsy and sample collection were performed by a wildlife veterinarian or veterinary pathologist. The information collected for each carcass is listed in Table 2, and these cases were included in the study’s data set. Sampling was performed under scientific licence (SL102578) issued by the Department of Planning, Industry and Environment, NSW Government. Ethics approval was not required for this study.

**Table 2:** Information collected from wild platypuses found deceased and presented to Taronga Zoo, Sydney, for necropsy and sample collection.

History	Carcass Assessment (Continued)
Date found	Total spur length (mm)
Date submitted	Spur sheath length (mm)
Submitter details (name, mobile number, email, affiliation)	Tail volume index (1–5) (see Table 3 for details)
Location of carcass (address/GPS coordinates)	Bill width (mm)
Habitat type where found	Bill length (with and without bill shield) (mm)
Recent weather events	Total body length (mm)
Evidence of pollution at location found	Tail length (mm)
**Carcass Assessment**	Tail width at midpoint (mm)
Microchip (present/absent)	Degree of decomposition (fresh; mild-moderate; advanced; mummified/skeletal)
Radiographs (subset of cases only)	Storage (fresh/chilled; frozen; other)
Weight	Gross necropsy
Sex	Histology (if carcass condition allowed)
Age	Additional testing (as indicated)

**Table 3:** Data extracted from medical records.

Variable	Values	Details of the Data Extraction Process
Local identification	The local identifier assigned by the submitter.	
Data submitter	Data submitter name (institution or individual).	
Data owner	The data owner name, where different to the data submitter.	
Submitter type	Database, private vet, researcher, state laboratory, university, wildlife hospital, zoo.	If a zoo facility had a free-ranging wildlife hospital, submitter type was classified as ‘zoo’ for zoo-housed platypus presentations and ‘wildlife hospital’ for wild platypus presentations.
Format	Digital—complete, digital—summary, paper.	‘Digital—complete’: records were provided entirely in a digital format. ‘Digital—summary’: only an electronic summary was available, as the original paper records were inaccessible. ‘Paper’: entirely paper-based records (including scanned paper records).
Ownership	Wild, zoo-housed.	‘Wild’: free-living prior to presenting to veterinarians or pathologists, including those that were temporarily housed at zoos or wildlife sanctuaries whilst undergoing treatment or rehabilitation. ‘Zoo-housed’: permanently housed at a zoo or wildlife sanctuary. Individuals that presented as ‘wild’ but were accessioned following treatment and became ‘zoo-housed’, were classified as ‘wild’ for the initial case presentation, and ‘zoo-housed for presentations subsequent to resolution of the presenting problems.
Date of presentation	Month, season, year.	Seasons: summer (December–February), autumn (March–May), winter (June–August), spring (September–November).
Location	Postcode, state/territory.	Postcode extrapolated from suburb. For zoo-housed platypuses, the address of the holding institution was considered the location.
Arrival status	Alive, found alive presented deceased, found dead.	
Sex	Male, female.	Sex determination in platypuses is possible on physical examination based on spur presence or absence, and morphology [4].
Age class	Nestling, juvenile, subadult, adult (Table 4) [4].	Age class was reclassified when either the age class was unspecified in the original record, but other supportive information was provided, or when there was a discrepancy between the descriptive information provided by the record’s author and the stated age class. Reclassification decisions were based on the following: - Based on provided supporting information. For example, descriptions or images of the bill [26], spur [4], or radiographs showing skeletal ossification [27]. - Individuals described as ‘orphaned’, ‘excavated from natal nest’, or similar, were classified as nestlings. - Individuals described as ‘juvenile-subadult’ were classified as juveniles. - Individuals were classified as juveniles if they weighed 35% less than the minimum recorded weight for an adult wild platypus of the relevant sex and geographic region *. This same criterion was also used to reclassify ‘sub-adult females’ (‘sub-adult’ is not recognised as an age class in female platypuses (Table 4)) as either juveniles or adults.
Reason for presentation	Abnormal behaviour and/or movement (inc. lethargy, weakness, lameness, circling, inappetence etc.); abnormal location (inc. beaches, estuaries, roads and backyards—noting these might be close to expected platypus habitat, such as a creek); entanglement; obvious wounds/skin abnormalities; excavated; planned drought rescue; during predation attempt; entrapment in a permanent human-made structure; died following capture for fieldwork; found dead; weight loss (zoo-housed only); diarrhoea (zoo-housed only); egg—failure to hatch (zoo-housed only); fertility investigation (zoo-housed only); routine physical examination (zoo-housed only).	Values assigned based on the provided history. The presentation of ‘entrapment in a permanent, human-made structure’ was designated for platypuses found stuck in drains, water tanks, nest boxes, or similar. It did not include fishing gear, such as yabby traps. ‘Entanglement’ did not distinguish between fishing gear and non-fishing rubbish. The ‘abnormal location’ value was assigned only if no other category was appropriate (e.g., if a platypus was found circling on a road, it would be classified as presenting for ‘abnormal behaviour and/or movement’, rather than ‘abnormal location’).
Tail volume index (TVI)	1 (emaciated)–5 (excellent) [3].	Where a body condition scoring system other than TVI had been used, or the written description provided did not match the stated TVI, a TVI was assigned that matched [3]. (Note that the TVI system published in [3] is reversed compared to previous TVI descriptions (inc. [26,28]). The system in [3] is consistent with other domestic animal and wildlife body condition scoring systems, where higher numbers indicate better body condition).
Outcome	Accessioned, unassisted death, found alive but died prior to presentation, euthanised, escaped—presumed dead, ongoing, procedure, released, resolved (zoo-housed only), sent to carer—lost to follow up.	The outcome was listed as ‘ongoing’ if the case was still in progress at the time the medical records were reviewed.
Diagnostics performed	Physical examination, haematology, biochemistry, imaging, urinalysis, faecal analysis, ante-mortem histology, post-mortem histology, microbiology, toxicology.	
Diagnostic confidence	High, low	Subjective assessment of the confidence in the reported diagnosis based on the information provided, such as whether diagnostic tests were performed and the reported results.
Morphological diagnosis, or clinical finding		Assigned by a specialist veterinary pathologist.
Body system associated with morphological diagnosis/clinical finding	Generalised, integumentary, musculoskeletal, nervous, cardiovascular, respiratory, digestive, urinary, endocrine, sensory, reproductive, haemolymphatic.	Each morphological diagnosis/clinical finding was exclusively assigned to one system, however an individual could have multiple systems impacted simultaneously by the same aetiological process (e.g., a single fishing gear entanglement presentation could result in morphological diagnoses involving the generalised, respiratory and integumentary body systems). Characterisation was performed by a specialist veterinary pathologist.
Aetiological category of morphological diagnosis/clinical finding. (Subcategories in brackets).	Congenital, degenerative, environmental, iatrogenic, indeterminate, infectious (bacterial, fungal, metazoan parasite, protozoan parasite, viral, unidentified), inflammatory, metabolic, neoplastic, egg death (undiagnosed), nutritional, reproductive, toxic, trauma (entrapment in a permanent human-made structure, fishing gear—hook or line, fishing gear—trap or net, predation, rubbish (non-fishing), self or conspecific, vehicular), undetermined.	Categorisation was performed by a specialist veterinary pathologist, based on the categories suggested by [22]. The trauma sub-categories differed from [22], in favour of categories considered to be more intuitive and relevant for platypuses. In diveregence from the recommendations of [22], an aetiological process could be classified as impacting on multiple body systems (e.g., a single infectious process could be classified as involving the neurological, respiratory, and integumentry systems). ‘Iatrogenic’ included fieldwork fatalities, excavated individuals, and husbandary errors. ‘Inflammatory’ was only applied if inflammation was considered the primary process (e.g., immune mediated disease). ‘Indeterminate’ was applied when the cause of disease remained unclear despite the application of all reasonable diagnostics. ‘Undetermined’ was applied when a diagnosis may have been reached if further reasonable diagnostics were performed (whilst aetiological categorisation was only applied to high diagnostic confidence cases, for some of these, further diagnostic testing could still have been undertaken).

* Field studies state that juveniles at emergence are 65–70% of their adult mass [29] and a study of zoo-housed platypuses showed nestlings at emergence were an average of 72% of the dam’s weight, and 66% of the sires [30]. In the current study, platypuses < 35% of the minimum adult weights recorded in [31] for the relevant sex and geographic region were classified as a juvenile, as it was considered more likely they were juveniles rather than a subadult or an emaciated adult. However, many juveniles will weigh more than this conservative value, and in these scenarios the erroneous classification of juveniles could not be detected, and subsequently corrected, using this criterion.

### 2.2. Record Review and Data Extraction

The record inclusion criteria were the medical records of all wild or zoo-housed platypuses in Australia. Medical records were defined as any clinical or pathology record produced by a clinical veterinarian or veterinary pathologist. Records were excluded based on the following criteria: healthy individuals captured during fieldwork or undergoing routine health examinations with no abnormalities detected; procedures performed on otherwise healthy individuals; autolysed carcasses or diagnostic testing (including blood tests or bacterial cultures) without accompanying information; anecdotal reports; records that were written without the direct involvement of a veterinarian or veterinary pathologist; or records where it was unclear if the platypus was wild or zoo-housed. Published case reports were only included when they accompanied the original medical record.

A standardised data extraction protocol and instrument were developed using Microsoft Excel (Microsoft 365 MSO, Redmond, WA, USA; Version 2301 Build 16.0.16026.20002). Record review and data extraction were performed by the first author. The study’s unit of observation was an individual platypus’s presentation to a clinician or pathologist (referred to as ‘presentation’ henceforth): each row in the data extraction instrument represented a single presentation by an individual platypus. For wild platypuses, presentations were considered separate if additional pathological processes developed whilst in care. For zoo-housed platypuses, presentations were considered separate if they met one or more of the following criteria: there were more than 12 months between the presentations; resolution of the problem was explicitly stated prior to its recurrence; two presentations occurred within 12 months but were considered separate aetiologies (e.g., sneezing noted for one week, and then an ulcer on the foot developed six months later). Where a single event resulted in multiple platypus presentations (e.g., multiple animals in a single abandoned trap), the individual platypuses involved were treated as a separate presentation. Each presentation was assigned a unique study identifier, composed of a unique whole number assigned to the individual platypus, and two decimal places reflecting the presentation number for that platypus.

The information extracted from records for each presentation is summarised in Table 3, including details of the data extraction procedures used for each variable (where required).

### 2.3. Data Analyses

Data were analysed in Microsoft Excel or SPSS Statistics (IBM, Armonk, NY, USA; Version 29.0.0.0 (241)). Most study variables were categorical, thus limiting statistical testing to non-parametric tests. Data analyses were primarily performed using descriptives, such as frequencies and chi-square analyses, with subsequent data visualisation. A *p*-value of <0.05 was considered statistically significant. Graphs and tables were produced in Microsoft Excel. Maps were created using Power BI (Microsoft Corporation, Redmond, WA, USA; Version 13.0.27228.39). Data were considered missing if information for a particular variable was not recorded on the medical record. Presentations with missing data were excluded from analyses where the missing data were relevant, unless otherwise stated (this resulted in a denominator that varied between analyses).

For wild platypuses, some analyses were performed only including the initial presentation, which is explicitly stated in the results section. Where specified, data from the ACT and NSW were combined, because the ACT is a small enclave within the much larger NSW. Where this occurred, the location was termed ‘NSW/ACT’. For all age-class analyses, the subadult age class (which is only recognised for male platypuses [4]; Table 4) was combined with the adult age class. This was done to improve consistency and comparability across sexes.

Where the objective of data analysis was to determine variables that could influence wild platypus presentations, only non-random reasons for presentation were included in analysis. The two presentation types that were considered random were ‘died following capture for fieldwork’ and ‘planned drought rescue’. It is specified in the results section when this occurred.

Only diagnoses with a ‘high’ diagnostic confidence (see Table 3 for explanation) were included in analyses of diagnoses. This study did not distinguish between incidental and clinically significant diagnoses due to challenges in interpreting clinical significance from retrospectively reviewed records. All diagnoses were reviewed by a Diplomate of the American College of Veterinary Pathologists (co-author CS).

## 3. Results

### 3.1. Medical Record Access

A total of 53 organisations or individuals were contacted regarding access to platypus medical records from wild or zoo-housed platypuses: Australia-wide = 1; NSW = 16; Queensland = 14; Tasmania = 9; South Australia = 6; Victoria = 6; ACT = 1. Of those contacted, 37.7% (20/53) had records that met the inclusion criteria and were shared in full, 47.2% (25/53) had no relevant records or provided records that did not meet the inclusion criteria. The remaining individuals or institutions (*n* = 8) either did not reply (*n* = 3), only partially shared records (*n* = 1), or indicated they had relevant records but did not share them (*n* = 4).

Records were provided for analyses in a complete digital format for 68.4% (262/383) of presentations. Almost one-fifth of presentations were provided in a paper format (18.0%, 69/383) across the entire study period (1991–2024), reducing only slightly over the most recent five-year period (2020–2024) covered by the study (13.2%; 12/90). Abbreviated digital summaries based on paper records that were no longer available were provided for 13.6% (52/383) of presentations.

### 3.2. Wild Platyuses

#### 3.2.1. Records

**Figure 1:**
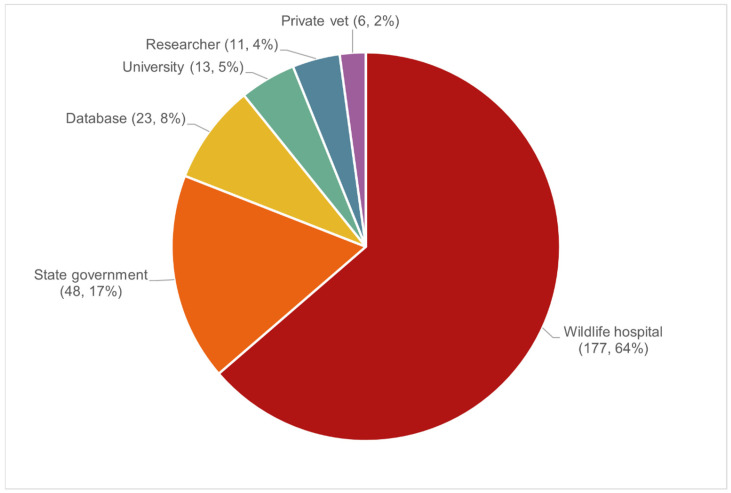
The number of wild platypus initial presentations (*n*, %) by submitter type (*n* = 278).

Medical records were received from 15 institutions, representing 6 submitter types (Figure 1).

**Figure 2:**
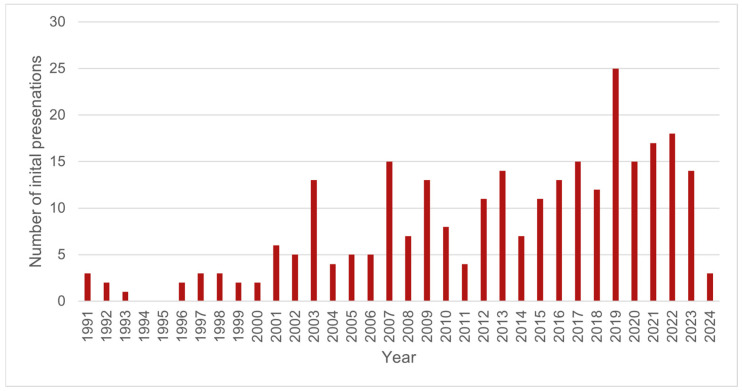
The number of wild platypus initial presentations by year (*n* = 278).

The records of 278 individual wild platypuses were reviewed, with a total of 293 presentations. Eight platypuses (2.9% (8/278) of individuals) presented more than once: six presented twice, one presented three times, and one presented eight times. Records spanned a 34-year period from 1991 to 2024. Figure 2 shows the number of wild platypus initial presentations by year.

#### 3.2.2. Location

**Figure 3:**
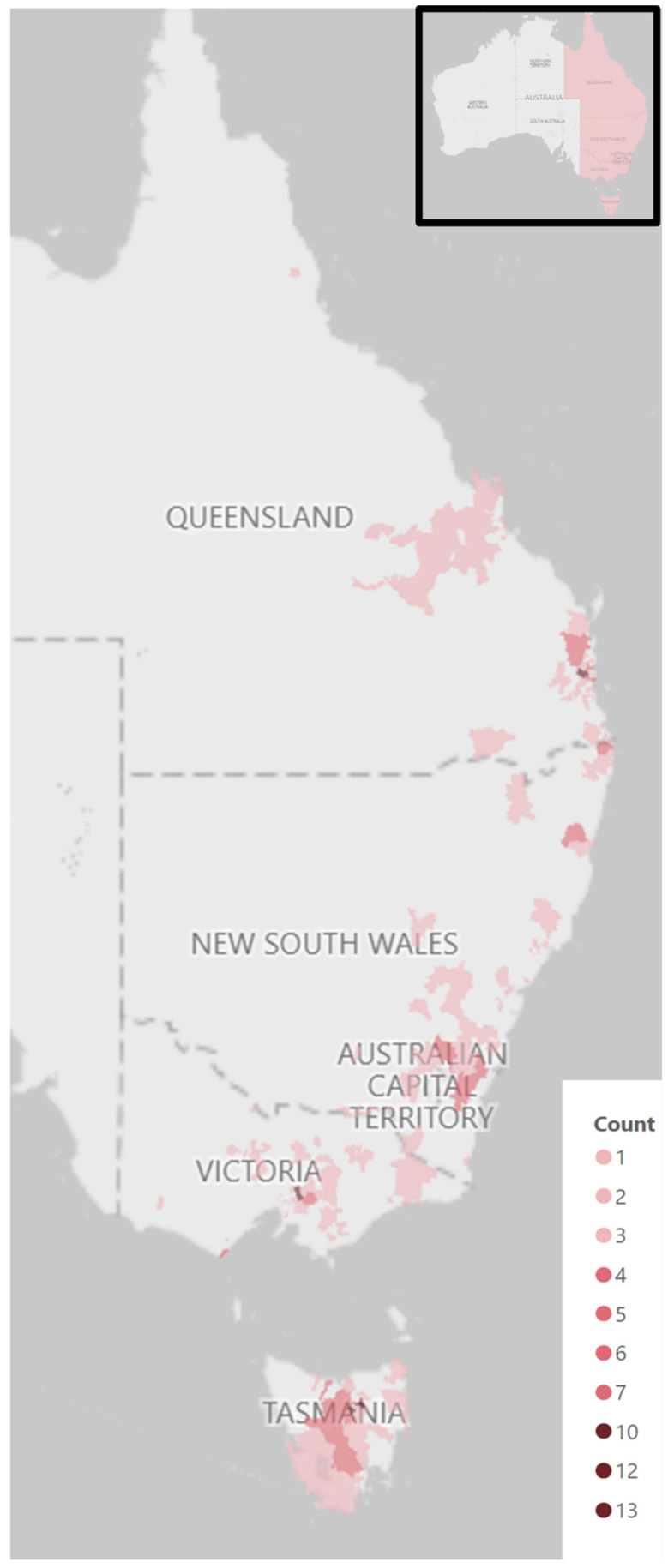
A heatmap of Australia’s east coast, showing the frequency of wild platypus initial presentations by postcode (*n* = 269). Inset—map of Australia.

There were no records of wild platypus presentations in South Australia. Combining NSW/ACT, the numbers of initial wild platypus presentations in the states/territories with presentations were approximately equal, with a mean of 68 (Queensland = 63; NSW/ACT = 70; Victoria = 73; Tasmania = 66). There were nine initial presentations in the ACT. These statistics were repeated with the inclusion of only non-random reasons for presentation (i.e., drought rescue and fieldwork deaths were excluded, *n* = 12). With this data subset, the number of wild platypus initial presentations remained approximately equal, with an average of 65 cases per state/territory. The number of cases in the ACT decreased to two. Figure 3 shows the number of individual platypus initial presentations by postcode. The number of presentations by postcode ranged from 0 to 13.

#### 3.2.3. Age

**Figure 4:**
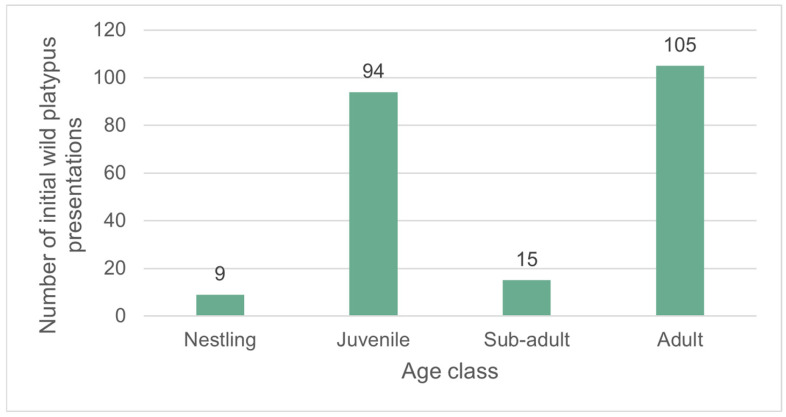
The total number of wild platypus initial presentations, by age class (*n* = 223).

Figure 4 displays the total number of wild platypus initial presentations by the four recognised platypus age classes: nestling, juvenile, sub-adult, and adult.

**Figure 5:**
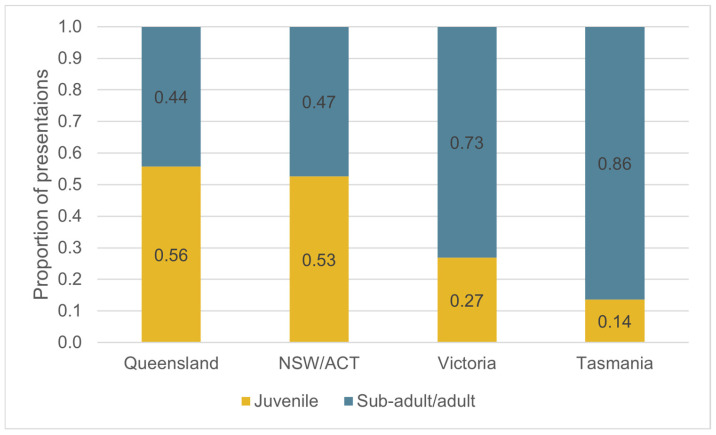
The proportion of wild platypus initial presentations, by juvenile and sub-adult/adult age class, and the state/territory of presentation, including only non-random reasons for presentation (nestling age group excluded due to small sample size) (*n* = 251).

The numbers of non-random initial presentations were compared between states/territories by age class. There was a significant difference in the proportion of juveniles and the sub-adult/adult age class presenting between the states/territories: χ^2^(3, 1; *n* = 251) = 33.8, *p* < 0.001 (Figure 5) (there were insufficient numbers of nestlings to include in the analysis). The northern states (Queensland and NSW) had a slight majority of juvenile presentations (Queensland: 55.7% juveniles, 34/61; NSW/ACT: 52.6% juveniles, 30/57). In contrast, the southern states (Victoria and Tasmania) had significantly fewer juvenile presentations than sub-adult/adult (Victoria: 26.9% juveniles; 18/67; Tasmania: 13.6% juveniles, 9/66).

#### 3.2.4. Season

**Figure 6:**
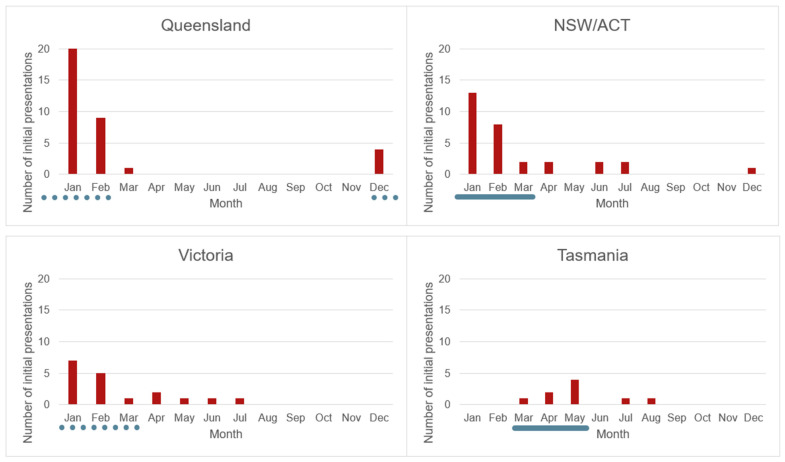
The month of wild juvenile initial presentations, by state/territory. Blue line: estimated time of nest emergence for platypuses in the relevant state/territory (solid line: timing of emergence is well-supported by field studies; dashed line: timing of emergence more approximate, with no confirmatory field studies) [1,3,4].

Including only non-random and initial reasons for presentation, the number of presentations varied by season. Summer was the most common season for wild platypuses to present (41.0% of presentations, 109/266) and winter the least common season (16.2% of presentations, 43/266). There was a significant relationship between season and the age class presenting (juvenile and sub-adult/adult age classes only; there were insufficient nestling presentations to include in the analysis): χ^2^(3, 1; *n* = 266) = 65.8, *p* < 0.001. The seasonality in presentations was driven by juvenile presentations, with 73.4% (69/94) of juvenile presentations occurring in summer. However, this varied between states (Figure 6), with no summer presentations of juveniles recorded in Tasmania: most juvenile Tasmanian presentations (7/9, 77.8%) occurred in autumn. The number of adult presentations was relatively consistent between seasons: summer 22.1% (36/163), autumn 27.0% (44/163), winter 20.9% (34/163), spring 30.1% (49/163).

#### 3.2.5. Sex

Including only non-random initial reasons for presentation where the sex was known, male platypuses were more likely to present than females overall: males represented 60.2% of presentations (133/221). This difference was greater in juveniles (males: 64.8% of presentations (57/88)) than in sub-adults/adults (males: 57.1% (76/133) of sub-adults/adults), however this difference was not statistically significant (χ^2^(1,1; *n* = 221) = 1.3, *p* = 0.26).

There was a significant relationship between sex and the state/territory of presentation: χ^2^(3, 1; *n* = 219) = 8.4, *p* = 0.04). This was greatest in NSW/ACT, where males comprised 73.9% (34/46) of wild platypus presentations. Queensland was the only state/territory that did not have a male bias in presentations (48.1% males, 25/52). Sex could not be determined from the history in a significant number of cases: 16.6% (46/278).

#### 3.2.6. Reason for Presentation

**Figure 7:**
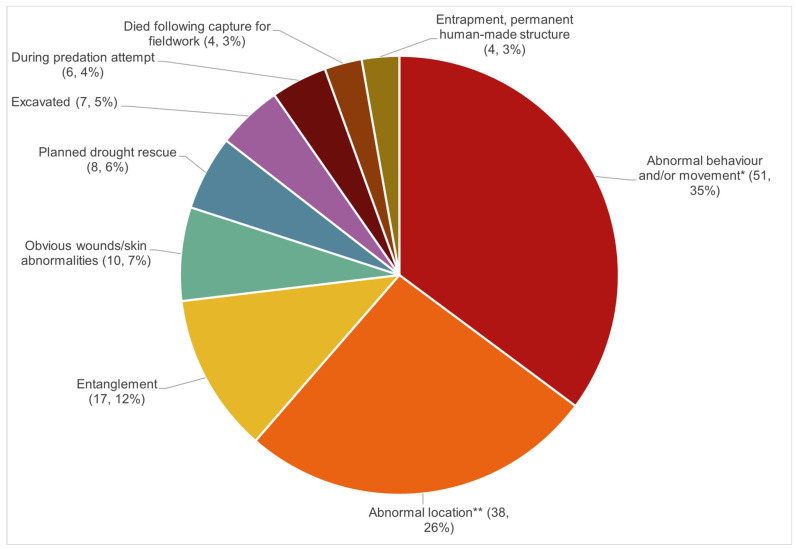
The reasons for alive wild platypus initial presentations (*n*, %), where sufficient history was available for categorisation (*n* = 145). * Includes lethargy, weakness, lameness, circling, inappetence etc. ** Includes beaches, estuaries, roads and backyards etc., noting these might be close to expected platypus habitats, such as a creek.

Platypuses were found deceased in 39.3% (94/239) of wild platypus initial presentations. Of those that were found alive, and where sufficient history was available to make a categorisation (*n* = 145), the reason for initial presentation is shown in Figure 7.

#### 3.2.7. Diagnostics Performed

Of 293 wild platypus presentations, 72.7% (213/293) had a morphological diagnosis or clinical finding for which there was a high level of confidence. Table 5 presents the diagnostic tests that were performed for these high diagnostic confidence presentations. The same presentation could have multiple diagnostic tests performed.

#### 3.2.8. Morphological Diagnoses/Clinical Findings

Table 6 displays the number of morphological diagnoses/clinical findings by body system and age class for the presentations with a high level of diagnostic confidence (*n* = 213). The five morphological diagnoses with the highest ratio of juvenile presentations to sub-adult/adult presentations were: anaemia (31.6%), heavy tick burden (subjective) (22.3%), enteritis (7.4%), splenic histiocytosis (7.4%), and emaciation (6.0%).

For the presentations with a high level of diagnostic confidence, diagnoses were also classified by aetiology: Table 7 displays the aetiological category and subcategory by age class and body system (*n* = 213). Collectively, the directly anthropogenic aetiologies (trauma from entrapment in a permanent human-made structure, trauma from fishing gear—hook or line, trauma from fishing gear—trap or net, trauma from rubbish (non-fishing), and trauma from vehicles) occurred in 44.1% all wild platypus presentations (94/213).

#### 3.2.9. Outcome

**Figure 8:**
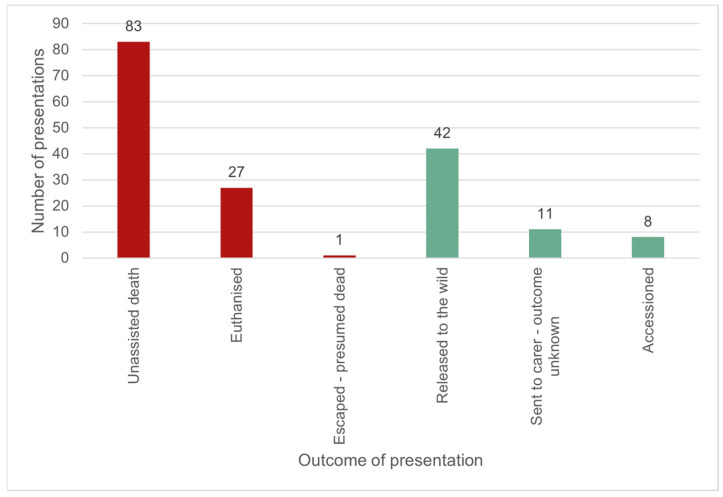
The outcome for live-presenting wild platypuses, for which the outcome was known (*n* = 172). Red—died prior to discharge; green—survived to discharge.

The outcomes for the 172 wild platypuses (non-random reasons for presentation only) that were presented to veterinarians alive, and for which the outcome was known, are summarised in Figure 8. Mortality (due to euthanasia, unassisted death, or escaped—presumed dead) was the outcome for 64.5% (111/172) of cases. The remaining 35.5% (61/172) of cases survived to discharge (defined as either being released to the wild, discharged to a carer (outcome unknown), or being accessioned into permanent zoo housing).

**Figure 9:**
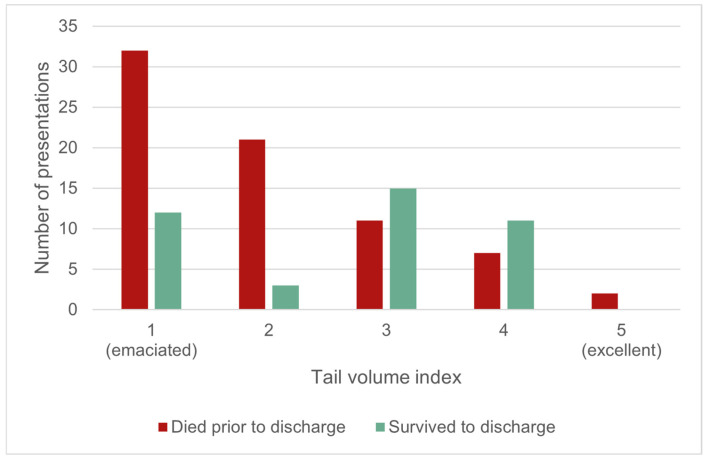
The outcome for live-presenting wild platypuses for which the outcome was known, by tail volume index (TVI) (*n* = 114).

There was a significant association between Tail Volume Index (TVI) and survival to discharge (χ^2^(4, 1; *n* = 114) = 18.6, *p* < 0.001; Figure 9). Animals that had an average to excellent TVI (TVI 3–5) had a survival rate of 56.5% (26/46); survival to discharge reduced to 22.1% (15/68) for animals with a poor or emaciated TVI (TVI 1–2). There was no statistically significant effect on survival to discharge between age class (nestling, juvenile, sub-adult/adult) (χ^2^(2, 1; *n* = 172) = 2.6, *p* = 0.28), or sex (χ^2^(1, 1; *n* = 150) = 0.2, *p* = 0.67).

### 3.3. Zoo-Housed Platypuses

The records of 40 individual zoo-housed platypuses were reviewed. Records spanned a 30-year period (1994–2023) and represented platypuses housed at nine institutions in four states (Queensland, NSW, Victoria, and Tasmania); there were no records that met the inclusion criteria received from South Australia or the ACT. There was a total of 90 presentations, with each platypus presenting between 1 and 7 times. The number of zoo-housed platypus presentations for a range of variables is displayed in Table 8.

The reasons zoo-housed platypuses presented are described in Table 9. Most zoo-housed platypuses (57.5%; 23/40) had at least one presentation for wounds or skin abnormalities.

Of the 90 presentations, 67.8% (61/90) had a diagnosis for which there was a high level of confidence. In Table 10, the diagnostic tests that were performed for these high diagnostic confidence cases are summarised.

Sixty-one presentations had morphological diagnoses/clinical findings with a high level of diagnostic confidence. These are displayed by body system in Table 11.

For the presentations with a high level of diagnostic confidence (*n* = 61), diagnoses were also classified by aetiology (Table 12).

Of the zoo-housed presentations for which an outcome could be determined (*n* = 88), resolution occurred in most cases (64.8%, 57/88); unassisted death occurred in 13.6% of cases (12/88); euthanasia occurred in 3.4% of cases (3/88); and 10.2% of cases were ongoing at the time the record was reviewed (9/88).

## 4. Discussion

This study revealed key trends in the reasons for the presentation of wild and zoo-housed platypuses to veterinarians and pathologists. For wild platypuses, amongst the most significant of these findings was a difference in the proportion of juvenile and sub-adult/adult presentations between states/territories, the timing of annual peaks in juvenile presentations, and the identification of previously unpublished causes of pathology in platypuses. For zoo-housed platypuses, the frequency with which presentations were attributable to wounds or skin abnormalities was a significant finding. These observations offer insights into wild platypus biology and highlight an important focus for zoo-housed platypus management, as described below.

An unexpected finding of the study was the significant difference in the proportion of wild juvenile and sub-adult/adult presentations between states (Figure 5). There was an apparent association between the proportion of juvenile presentations and latitude, with juveniles representing a minority of presentations in the southern states of Victoria and Tasmania, but a slight majority of all presentations in the northern states of Queensland and NSW. This is despite the juvenile age class likely only representing 6–16% of the wild population age structure: platypuses are classified as juveniles for ~8 months (from nest emergence—at ~4 months—to 12 months of age) [4], and the average wild platypus lifespan is 6–15 years [1]. Only in Tasmania was the ratio of juvenile to sub-adult/adult presentations (13.6%) proportional to the likely number of juveniles in the overall population.

The disproportionate number of juvenile presentations in the northern states can be interpreted alongside another of this study’s observations: that juvenile presentations were tightly clustered around the expected time of nest emergence in their respective states (Figure 6). Juvenile nest emergence occurs in late January to early March (summer to early autumn) on the mainland, and approximately two months later (late March to early May, i.e., autumn) in Tasmania [1,4]. Emergence from the nest corresponds with weaning, and the limited available evidence suggests weaning is abrupt in platypuses [32] and thus is likely to be a significant stressor for juveniles.

It is likely that recently weaned juvenile platypuses have less resilience than adults, and thus if they experience stressors beyond the baseline challenge of weaning they may rapidly fail to cope. One hypothesis for the latitudinal difference in the proportion of wild juvenile and sub-adult/adult presentations is that platypuses in the northern states are experiencing greater stressors during this vulnerable weaning period than those in the south. These stressors could include: reduced food availability (e.g., due to lower availability of suitable macroinvertebrate prey species); increased competition with other aquatic species (e.g., variations in fish numbers and/or species across the range); increased extreme weather events (such as flooding) that occur more frequently in summer in northern Australia; or other environmental stressors, such as high ambient temperatures. Juveniles may be physiologically less heat tolerant, or less able to employ behavioural thermoregulatory mechanisms. For example, they may be less capable than adults of finding or competing for suitable burrows [33,34], or competition with adults may force them to increase overland travel or to forage during warmer parts of the day. If increased juvenile presentations in the north is reflective of reduced juvenile survival in their northern range, it is possible this could impact population recruitment and thus have implications for platypus conservation.

The observation that the peak in juvenile presentations corresponds to the timing of nest emergence and weaning refutes a previously proposed hypothesis that juvenile platypuses most commonly present at the time of dispersal [9,26,35]. It was suggested that platypuses frequently present to clinics as dispersing juveniles in poor body condition due to insufficient food intake. The dispersal behaviour of platypuses is a challenging area to research and remains poorly understood [32]; however, available evidence suggests it does not occur immediately after juveniles leave the nest [32,36,37]. This delayed dispersal strategy potentially benefits weaning juveniles by ensuring they remain in an environment with known access to resources during the vulnerable post-emergence period [32]. Thus, the tight association observed in this study between the peak of juvenile presentations and expected time of nest emergence supports the theory that juvenile presentations are occurring at the time of weaning, rather than dispersal.

Consistent with previous studies [14,15,16], anthropogenic aetiologies were common in wild platypuses, occurring in almost half of all wild platypus presentations (Table 7). Although this study did not distinguish between the species responsible for predation events, it is likely that a significant proportion are attributable to non-native species (particularly domestic dogs and foxes), and thus many of these predation events may also by extension be considered anthropogenic in origin. However, compared to previous morbidity and mortality studies in platypuses, many additional disease processes were identified. This finding is likely to be attributable to the higher sample size (relative to [14]), and the higher level of diagnostic investigation that had been performed by clinical veterinarians and veterinary pathologists for many of the presentations (relative to the cases reported in [15,16]). Across wild and zoo-housed platypuses, previously unreported diagnoses that were identified through this retrospective review process include round cell neoplasia, neural angiostrongyliasis, and iron deficiency anaemia.

A concerning finding in zoo-housed platypuses was the frequency of wounds or skin abnormalities, with 57.5% of individuals having at least one presentation for this reason, and 70.5% of presentations diagnosed with an aetiology related to the integumentary system (Table 12). Of those skin abnormalities for which an underlying aetiology could be determined, the majority were fungal infections. There are sporadic reports of skin lesions in zoo-housed platypuses in the literature [26,38], but the frequency of presentations in this data set suggests that underlying husbandry practices should be investigated as these may be predisposing to secondary infections. Contributing factors that should be considered include nutritional deficiencies in the zoo-housed platypus diet, ultraviolet lighting requirements (zoo-housed platypuses are frequently held indoors without access to sunlight), abrasive surfaces in provided habitats, and water quality.

Retrospective reviews are susceptible to errors and biases, including those resulting from errors during the process of extracting data from clinical records, missing data, lack of homogeneity due to variability in the quality and quantity of original records, and loss to follow up [19,21,23]. All these limitations were present in this study, but attempts were made to minimise the impact on results and conclusions by applying the STROBE guidelines [23] and the recommendations made by [22], where possible. As described in the methodology, there were intentional adaptations of the recommendations made by [22]. Most significantly, in the present study, a single aetiological process could be classified as impacting on multiple body systems in one presentation. This was performed to provide a more comprehensive insight into the pathogenesis and impact of disease processes, thus potentially assisting clinicians with targeted decision making and thereby improving outcomes.

A limitation of the study is that there is expected to be significant variation in the level of clinician knowledge, skills and experience with platypuses, including with the accepted systems for assigning age, sex, and body condition. The level of record accuracy is challenging to assess in the review process but could result in some data inaccuracies. It’s possible that the reason for the slight male bias in wild platypus presentations for all states/territories (other than Queensland) is due to a lack of clinician confidence with sexing platypuses. The sex of the platypus was missing from the reviewed records in 16.6% of cases, and it is possible that clinicians are more confident with classifying a platypus with spurs as male, but more reluctant to assign sex to a spurless (female) platypus. Other data inaccuracies could result because some morphological diagnoses or clinical findings (such as fractures, emaciation, or entanglement and entrapment in fishing gear) can be made more readily than others, and this could falsely elevate the apparent proportion of these relative to more subtle processes. Additionally, the sub-category of ‘trauma—unknown/other’, likely includes some cases from other sub-categories (such as ‘trauma—vehicular’).

In addition to the above caveats, some results from this study should be interpreted with caution, in particular, survival to discharge for wild platypuses, and the number of presentations by year for both wild and zoo-housed platypuses. The number of presentations by year is complicated by inconsistent sampling effort: institutions were operating over different time periods; older records were less likely to be accessible; and the prolonged, three-year record submission period meant that records for presentations that occurred between August 2021 and September 2024 may not have been submitted by all institutions. Regarding survival to discharge, the gold standard for determining survival is post-release monitoring, but, to date, there is only a single known case of post-release monitoring occurring for a rehabilitated platypus [33]. Using proxies for ‘true’ survival (such as ‘survival to discharge’) is likely to underestimate mortality. Whilst it was not quantified as part of the review, qualitatively, many of the platypuses that were discharged to carers were in states of considerable compromise and it is considered unlikely all survived. Similarly, several platypuses were released a short time after presenting despite evidence of significant compromise on physical examination and it is probable at least some died following release. Thus, the true survival rate for platypuses presented alive to veterinarians is likely to be lower than the survival to discharge value of 35.5% calculated in this study.

A strong recommendation from this study is that medical records should be both permanent and digital, and include fundamental data, such as age class, sex, and outcome. A significant number of medical records reviewed for this study were in a paper format, even in the most recent five-year period covered by the study (2020–2024). This substantially increased the workload involved in performing the review, necessitating interstate travel to retrieve records, and requiring the deciphering of poor-quality handwriting. Furthermore, fundamental data (such as sex, age, and TVI) were not recorded for a considerable number of cases. Digitalising records and standardising the inclusion of fundamental information will reduce the barriers to performing effective retrospective reviews and maximise the insights gained through the review process. Doing so will also allow the application of emerging retrospective record review techniques, such as text-mining, which have the potential to revolutionise the process [39].

## 5. Conclusions

The retrospective review provides a valuable methodology for improving our understanding of the health and disease of wildlife species that are relatively infrequently encountered by veterinarians and pathologists. Its application to platypus medical records has identified unexpected trends and priorities for future research, including establishing why the juvenile age class presents with disproportionate frequency in northern states, and why zoo-housed platypuses so frequently present with skin lesions. Directing future research towards these topics will ultimately assist in maximising conservation outcomes for wild platypuses and improving the welfare of zoo-housed platypuses.

## Figures and Tables

**Table 1:** Criteria for identifying the organisations and individuals most likely to hold platypus medical records. (ZIMS, Zoological Information Management System [24]; WHA, Wildlife Health Australia [25]).

Organisation/Individual Type	Criteria
Zoo or wildlife sanctuary	Listed on ZIMS [24] as holding platypuses.
	Internet search indicates currently holds, or has historically held, platypuses.
	Member of WHA’s zoo-based wildlife health surveillance program and located within the platypus’s range. *
Private veterinary clinic	Member of the WHA’s sentinel clinic wildlife health surveillance program and located within the platypus’s range.
	Anecdotally reported to have a high wildlife case load and located within the platypus’s range.
	Considered likely to have previously treated platypuses (based on social media or anecdotal reports).
Wildlife hospital	Located within the platypus’s range.
University	Member of the WHA’s university-based wildlife health surveillance program and located within the platypus’s range.
Researcher	Known to have conducted platypus fieldwork.
State/territory government	Within the platypus’s range.
Wildlife health database	Established databases of Australian wildlife health.
Individual wildlife veterinarian	Wildlife veterinarians not affiliated with an organisation at the time of contact but considered likely to have historically been involved with platypus health care.
Other (inc. wildlife rehabilitators and wildlife rehabilitation organisations)	Contact suggested by other organisations or individuals involved with platypuses.

* Additional details about WHA’s surveillance programs can be found at https://wildlifehealthaustralia.com.au/Our-Work/Surveillance (accessed on 25 May 2021).

**Table 4:** Recognised platypus age classes and the age at which a platypus is classified into each, by sex [4].

Age Class	Male	Female
Nestling	Male and female: 0 (hatching)–~3 months (until first emergence from the nest)	
Juvenile	4–12 months	4–9 months
Sub-adult	12–24 months	Age class not recognised
Adult	>24 months	Females: >9 months

**Table 5:** Diagnostic tests performed for wild platypus presentations (high diagnostic confidence presentations only) (*n* = 213).

Diagnostic Test	Number of Presentations Performed for
Gross necropsy	125
Ante-mortem physical examination	101
Histology (post-mortem)	86
Haematology	59
Biochemistry	57
Diagnostic imaging	47
Microbiology	31
Urinalysis or faecal analysis	20
Histology (ante-mortem)	5
Toxicology	5

**Table 6:** The number of morphological diagnoses/clinical findings in wild platypuses (high diagnostic confidence presentations only), by body system and age class (nestling, juvenile, sub-adults/adults) (*n* = 213). **Bold:** body system. *Italicised text, first row of each body system:* the combined results for each body system. (Brackets): the percentage of total presentations for that category. Diagnoses with <3 presentations are grouped as ‘other’; * = the expanded list of ‘other’ diagnoses (*n*). Body systems, and within that, the morphological diagnoses/clinical findings, are ordered from the highest to lowest number of presentations. Note that a single presentation could have multiple diagnoses, including within the same body system.

Body System	Morphological Diagnosis/Clinical Finding	Nestlings (*n* = 7)	Juveniles (*n* = 72)	Sub-Adults/ Adults (*n* = 134)	Total (*n* = 213)
**Generalised**					
	*Generalised, combined*	*5 (71.4%)*	*46 (63.9%)*	*89 (66.4%)*	*140 (65.7%)*
	Emaciation	0 (0%)	32 (44.4%)	10 (7.5%)	42 (19.7%)
	Trauma, predation	0 (0%)	5 (6.9%)	23 (17.2%)	28 (13.2%)
	Trauma, fishing gear	0 (0%)	4 (5.6%)	20 (14.9%)	24 (11.3%)
	Trauma, unspecified	0 (0%)	7 (9.7%)	14 (10.5%)	21 (9.9%)
	Trauma, vehicular	0 (0%)	1 (1.4%)	14 (10.5%)	15 (7.0%)
	Trauma, rubbish (non-fishing)	0 (0%)	1 (1.4%)	11 (8.2%)	12 (5.6%)
	Orphaned	5 (71.4%)	0 (0%)	0 (0%)	5 (2.4%)
	Sepsis	0 (0%)	2 (2.8%)	2 (1.5%)	4 (1.9%)
	Other (5 additional diagnoses *)	0 (0%)	4 (5.6%)	3 (2.2%)	7 (3.4%)
* Dehydration (2), died following entrapment in a permanent human-made structure (2), hyperthermia (1), neoplasia (1), suspected electrocution (1).					
**Integumentary**					
	*Integumentary, combined*	*3 (42.9%)*	*27 (37.5%)*	*43 (32.1%)*	*73 (34.3%)*
	Dermatitis	1 (14.3%)	9 (12.5%)	9 (6.7%)	19 (8.9%)
	Heavy tick burden (subjective)	0 (0%)	12 (16.7%)	1 (0.8%)	13 (6.1%)
	Wounds, rubbish entanglement	0 (0%)	1 (1.4%)	11 (8.2%)	12 (5.6%)
	Cutaneous ulceration	0 (0%)	0 (0%)	10 (7.5%)	10 (4.7%)
	Wounds, unknown cause	0 (0%)	4 (5.6%)	4 (3.0%)	8 (3.8%)
	Wounds, fishing gear	0 (0%)	1 (1.4%)	7 (5.2%)	8 (3.8%)
	Folliculitis/furunculosis	0 (0%)	6 (8.3%)	0 (0%)	6 (2.8%)
	Cutaneous nematodiasis	0 (0%)	0 (0%)	4 (3.0%)	4 (1.9%)
	Superficial abrasions	1 (14.3%)	2 (2.8%)	1 (0.8%)	4 (1.9%)
	Contusions	0 (0%)	0 (0%)	3 (2.2%)	3 (1.4%)
	Other (13 additional diagnoses *)	2 (28.6%)	6 (8.3%)	6 (4.5%)	14 (6.6%)
* Nail/spur keratinopathy (2), alopecia (unclear if underlying dermatitis) (1), crural adenitis (1), dermal oedema (1), fibrosis (1), fractured/missing spur (1), hyperkeratosis (1), mass: unknown aetiology (1), parakeratotic hyperkeratosis (1), pericloacal papules (1), pruritis (1), thickening of skin (1), ulceration (1).					
**Digestive**					
	*Digestive, combined*	*2 (28.6%)*	*30 (41.7%)*	*29 (21.6%)*	*61 (28.6%)*
	Gastrointestinal coccidia present	0 (0%)	13 (18.1%)	9 (6.7%)	22 (10.3%)
	Enteritis	1 (14.3%)	8 (11.1%)	2 (1.5%)	11 (5.2%)
	Hepatic necrosis	1 (14.3%)	5 (6.9%)	5 (3.7%)	11 (5.2%)
	Hepatitis	0 (0%)	3 (4.2%)	2 (1.5%)	5 (2.4%)
	Hepatic congestion	0 (0%)	1 (1.4%)	3 (2.2%)	4 (1.9%)
	Hepatic lipidosis	0 (0%)	4 (5.6%)	0 (0%)	4 (1.9%)
	Cholangiohepatitis	0 (0%)	0 (0%)	3 (2.2%)	3 (1.4%)
	Other (30 additional diagnoses *)	2 (28.6%)	14 (19.4%)	18 (13.4%)	34 (16.0%)
* Alimentary helminth (2), gastritis (2), gastroenteritis (2), vacuolated hepatocytes (2), centrilobular degeneration (1), changes consistent with intestinal stasis (1), cloacitis (1), colitis (1), enteric coccidia (1), enteric trematodiasis (1), enteritis with intra-mucosal metazoan parasites (1), exocrine acinar atrophy (1), fatty degeneration of the liver (1), flagellates in intestinal crypts (1), glossitis (1), hepatic accumulations of plasma cells (1), hepatic carcinoid (1), hepatic fibrosis (1), hepatic karyomegaly (1), hepatocellular atrophy (1), hepatopathy (1), impacted cheek pouch (1), lipoid vacuolation of enterocytes (1), oral ulceration (1), pancreatic necrosis (1), periacinar cuffing with inflammatory cells (1), periodontitis (1), salivary gland polyoma-like intranuclear inclusion bodies (1), stomach ulceration (1), vacuolated hepatocytes (1).					
**Respiratory**					
	*Respiratory, combined*	*1 (14.3%)*	*20 (27.8%)*	*38 (28.4%)*	*59 (27.7%)*
	Pulmonary congestion	1 (14.3%)	4 (5.6%)	8 (6.0%)	13 (6.1%)
	Pulmonary oedema	0 (0%)	3 (4.2%)	9 (6.7%)	12 (5.6%)
	Pneumonia	0 (0%)	5 (6.9%)	4 (3.0%)	9 (4.2%)
	Drowning, fishing gear	0 (0%)	2 (2.8%)	6 (4.5%)	8 (3.8%)
	Pneumonia—fungal	0 (0%)	0 (0%)	4 (3.0%)	4 (1.9%)
	Pulmonary interstitial reaction	0 (0%)	2 (2.8%)	2 (1.5%)	4 (1.9%)
	Pneumonia—bacterial	0 (0%)	3 (4.2%)	0 (0%)	3 (1.4%)
	Other (9 additional diagnoses *)	0 (0%)	5 (6.9%)	7 (5.2%)	*12 (5.6%)*
* Pneumonia—aspiration (2), pneumonia (pulmonary inflammation) (2), pulmonary haemorrhage (2), bleeding from nares (1), drowning (fieldwork) (1), pneumonia—protozoan (1), pulmonary fibrosis (1), pyothorax (1), tracheal oedema (1).					
**Musculoskeletal**					
	*Musculoskeletal, combined*	*1 (14.3%)*	*12 (16.7%)*	*31 (23.1%)*	*44 (20.7%)*
	Fracture/s	1 (14.3%)	10 (13.9%)	29 (21.6%)	40 (18.8%)
	Other (4 additional diagnoses *)	0 (0%)	3 (4.2%)	2 (1.5%)	5 (2.4%)
* Myositis (2), muscle oedema (1), parasitic myositis (1), serous atrophy of adipose tissue (1).					
**Urinary**					
	*Urinary, combined*	*1 (14.3%)*	*11 (15.3%)*	*25 (18.7%)*	*37 (17.4%)*
	Interstitial mononuclear nephritis	0 (0%)	0 (0%)	13 (9.7%)	13 (6.1%)
	Interstitial congestion of the kidney	1 (14.3%)	1 (1.4%)	2 (1.5%)	4 (1.9%)
	Nephropathy	0 (0%)	1 (1.4%)	3 (2.2%)	4 (1.9%)
	Renal tubular degeneration and necrosis	0 (0%)	1 (1.4%)	3 (2.2%)	4 (1.9%)
	Other (17 additional diagnoses *)	0 (0%)	8 (11.1%)	14 (10.5%)	22 (10.3%)
* Cystitis (2), glomerulitis (2), lipoid vacuolation of renal tubular epithelium (2), nephrocalcinosis (2), renal lipidosis (2), adenoviral infection of collecting tubules (1), bilirubin pigment in renal tubular epithelium (1), fine brown granules in the convoluted tubules (1), hyaline granule deposits in renal tubular epithelium (1), intraluminal pigment accumulation (1), nephritis (1), renal basophilic intranuclear inclusion bodies (1), renal coccidiosis (1), renal granuloma (1), renal tubular degeneration without necrosis (1), single kidney (1), vacuolations—unspecified (1).					
**Haemolymphatic**					
	*Haemolymphatic, combined*	*0 (0%)*	*16 (22.2%)*	*11 (8.2%)*	*27 (12.7%)*
	Splenic extramedullary haematopoiesis	0 (0%)	9 (12.5%)	6 (4.5%)	15 (7.0%)
	Splenic histiocytosis	0 (0%)	4 (5.6%)	1 (0.8%)	5 (2.54%)
	Splenic lymphoid hyperplasia	0 (0%)	3 (4.2%)	1 (0.8%)	4 (1.9%)
	Splenic necrosis	0 (0%)	1 (1.4%)	2 (1.5%)	3 (1.4%)
	Other (8 additional diagnoses *)	0 (0%)	4 (5.6%)	7 (5.2%)	11 (5.2%)
* Bone marrow haematopoietic hyperplasia (2), splenic lymphocytolysis (2), splenic plasma cell hyperplasia (2), splenic round cell tumour (2), bone marrow erythroid hyperplasia with left shift (1), bone marrow granulocytic hyperplasia (1), splenic lymphoid depletion (1).					
**Cardiovascular**					
	*Cariovascular, combined*	*0 (0%)*	*18 (25.0%)*	*8 (6.0%)*	*26 (12.2%)*
	Anaemia	0 (0%)	17 (23.6%)	1 (0.8%)	18 (8.5%)
	Other (7 additional diagnoses *)	0 (0%)	2 (2.8%)	7 (5.2%)	9 (4.2%)
* Erythrocytic parasitism (2), myocarditis (2), cardiac round cell tumour (1), endocarditis (1), epicarditis (1), haemosiderosis of the subendocardial myocardium (1), myocardial necrosis (1).					
**Nervous**					
	*Nervous, combined*	*0 (0%)*	*4 (5.6%)*	*9 (6.7%)*	*13 (6.1%)*
	Congestion of meningeal vasculature	0 (0%)	3 (4.2%)	0 (0%)	3 (4.4%)
	Other (8 additional diagnoses *)	0 (0%)	1 (1.4%)	10 (7.5%)	11 (5.2%)
* Encephalomalacia (2), meningoencephalitis—unknown cause (2), meningoencephalitis—*Angiostrongylus cantonensis* (2), meningitis (1), meningoencephalitis—*Toxoplasma gondii* (1), neuronal lipofuscinosis (1), perivascular oedema (1), proprioceptive deficits (1).					
**Endocrine**					
	*Endocrine, combined*	*0 (0%)*	*5 (7.0%)*	*5 (3.7%)*	*10 (4.7%)*
	Adrenal cortical hyperplasia	0 (0%)	2 (2.8%)	2 (1.5%)	4 (1.9%)
	Thyroid hyperplasia	0 (0%)	3 (4.2%)	0 (0%)	3 (1.4%)
	Other (5 additional diagnoses *)	0 (0%)	2 (2.8%)	3 (2.2%)	5 (2.4%)
* Accelerated thymic atrophy (1), adrenal gland congestion (1), adrenal karyomegaly (1), hypothyroidism (1), thymus lymphocytolysis (1).					
**Sensory**					
	*Sensory, combined*	*0 (0%)*	*0 (0%)*	*2 (1.5%)*	*2 (1.0%)*
	Other (2 diagnoses *)	0 (0%)	0 (0%)	1 (0.8%)	2 (0.9%)
* Basophilic mineral deposit (1), lenticular degeneration (1).					
**Reproductive**					
	*Reproductive, combined*	*0 (0%)*	*0 (0%)*	*1 (0.8%)*	*1 (0.5%)*
	Other (1 diagnosis *)	0 (0%)	0 (0%)	1 (0.8%)	1 (0.5%)
* Mammary lobular hyperplasia, with lactation (1).					

**Table 7:** The aetiological category and subcategory for diagnoses in wild platypuses (high diagnostic confidence presentations only), by age class and body system (*n* = 213). Total % calculated as a percentage of the total number of presentations (i.e., 213). Categories are listed in alphabetical order.

		Body System												
Category	Sub-Category	Generalised	Integumentary	Musculoskeletal	Nervous	Cardiovascular	Respiratory	Digestive	Urinary	Endocrine	Sensory	Reproductive	Haemolymphatic	Total by Category
Congenital		0	0	0	0	0	0	0	1	0	0	0	0	1 (0.5%)
Degenerative		0	0	0	1	0	0	1	0	0	1	0	0	3 (1.4%)
Environmental		1	0	0	0	0	0	0	0	0	0	0	0	1 (0.5%)
Iatrogenic		0	1	0	0	0	1	0	0	0	0	0	0	2 (0.9%)
Indeterminate		0	3	0	4	1	3	9	1	1	0	0	1	23 (10.8%)
Infectious	Bacterial	3	3	1	0	0	6	5	0	0	0	0	0	18 (8.5%)
Infectious	Viral	0	0	0	0	0	0	0	1	0	0	0	0	1 (0.5%)
Infectious	Fungal	0	25	1	0	0	6	1	0	0	0	0	0	33 (15.5%)
Infectious	Metazoan parasite	0	17	1	2	6	0	5	0	0	0	0	2	33 (15.5%)
Infectious	Protozoan parasite	0	0	0	1	3	1	30	1	0	0	0	1	37 (17.4%)
Infectious	Unidentified	0	0	0	0	0	2	2	2	0	0	0	0	6 (2.8%)
Inflammatory		0	1	0	0	0	1	0	0	0	0	0	0	2 (0.9%)
Metabolic		0	0	0	0	0	0	0	0	0	0	0	0	0 (0%)
Neoplastic		3	0	0	0	1	0	1	0	0	0	0	2	7 (3.3%)
Egg death	Undiagnosed	0	0	0	0	0	0	0	0	0	0	0	0	0 (0%)
Nutritional		42	0	2	0	0	0	8	4	0	0	0	0	56 (26.3%)
Reproductive		0	0	0	0	0	0	0	0	0	0	1	0	1 (0.5%)
Toxic		0	0	0	0	0	0	2	0	0	0	0	0	2 (0.9%)
Trauma	Other/ unknown	22	17	15	0	0	3	0	0	0	0	0	0	57 (26.8%)
Trauma	Entrapment in a permanent, human-made structure	2	0	0	0	0	0	0	0	0	0	0	0	2 (0.9%)
Trauma	Fishing gear—hook or line	12	7	0	0	0	5	0	0	0	0	0	0	24 (11.3%)
Trauma	Fishing gear—trap or net	12	0	0	0	0	4	0	0	0	0	0	0	16 (7.5%)
Trauma	Predation	28	2	12	0	0	0	0	0	0	0	0	0	42 (19.7%)
Trauma	Rubbish (non-fishing)	12	12	0	0	0	0	0	0	0	0	0	0	24 (11.3%)
Trauma	Self or conspecific	0	0	0	0	0	0	0	0	0	0	0	0	0 (0%)
Trauma	Vehicular	15	0	12	0	0	1	0	0	0	0	0	0	28 (13.2%)
Undetermined		1	13	1	3	12	10	19	24	3	0	0	6	92 (43.2%)
Total by body system		153 (71.8%)	101 (47.4%)	45 (21.1%)	11 (5.2%)	23 (10.8%)	43 (20.2%)	83 (39.0%)	34 (16.0%)	4 (1.9%)	1 (0.5%)	1 (0.5%)	12 (5.6%)	

**Table 8:** The number of zoo-housed platypus presentations for the variables of sex, age, state/territory, and season (*n* = 90).

Variable	Category	Number of Presentations	% of Presentations
Sex	Female	40	44.4
	Male	49	54.4
	Unknown *	1	1.1
Age	Nestling	1	1.1
	Juvenile	3	3.3
	Sub-adult/adult	86	95.6
State housed	Queensland	9	10.0
	NSW	48	53.3
	ACT	0	0
	Victoria	27	30.0
	Tasmania	6	6.7
	South Australia	0	0
Season of presentation	Summer	23	25.6
	Autumn	19	21.1
	Winter	28	31.1
	Spring	20	22.2

* autolysed nestling.

**Table 9:** The reasons for zoo-housed platypus presentations (*n* = 90).

Reason for Presentation	Number of Presentations	% of Presentations
Obvious wounds/skin abnormalities	50	55.6
Abnormal behaviour and/or movement (inc. lethargy, weakness, lameness, circling, inappetence)	25	27.8
Found dead	5	5.6
Weight loss	5	5.6
Diarrhoea	1	1.1
Egg—failure to hatch	1	1.1
Fertility investigation	1	1.1
Routine physical examination	1	1.1
Unknown *	1	1.1

* histology results provided without history.

**Table 10:** Diagnostic tests performed for zoo-housed platypus presentations (high diagnostic confidence presenations only) (*n* = 61).

Diagnostic Test	Number of Presentations Performed for
Ante-mortem physical examination	46
Microbiology	25
Haematology	21
Biochemistry	20
Gross necropsy	13
Histology (post-mortem)	12
Histology (ante-mortem)	11
Diagnostic imaging	6
Urinalysis or faecal analysis	3
Toxicology	0

**Table 11:** The number of morphological diagnoses/clinical findings in zoo-housed platypuses (high diagnostic confidence presentations only) by body system (*n* = 61). **Bold:** body system. *Italicised text, first row of each body system:* the combined results for each body system. (Brackets): the percentage of total presentations for that category. Diagnoses with <3 presentations are grouped as ‘other’; * = the expanded list of ‘other’ diagnoses (*n*). Body systems, and within that, the morphological diagnoses/clinical findings, are ordered from the highest to the lowest number of presentations. Note that a single presentation could have multiple diagnoses, including within the same body system.

Body System	Morphological Diagnosis/Clinical Finding	Number of Presentations with the Diagnosis (*n* = 61)	% of Presentations (*n* = 61)
**Integumentary**			
	*Integumentary, combined*	*46*	*75.4*
	Alopecia without underlying dermatitis	17	27.9
	Dermatitis	12	19.7
	Cutaneous ulceration	6	9.8
	Fractured/missing spur	4	6.6
	Nail/spur keratitis	3	4.9
	Other (17 additional diagnoses *)	22	36.1
* Acanthosis (2), alopecia (unclear if underlying dermatitis) (2), barbering (2), cutaneous necrosis (2), wounds—unknown cause (2), bacterial venom gland adenitis (1), cutaneous papilloma (1), cyst/pustule (1), erythema of spur base (1), erythema of bill/feet (1), folliculitis/furunculosis (1), mass: unknown aetiology (1), nail dysplasia (1), nail keratinopathy (1), parakeratotic hyperkeratosis (1), spur keratinopathy (1), wounds (conspecific) (1).			
**Digestive**			
	*Digestive, combined*	*9*	*14.8*
	Enteritis	5	8.2
	Other (6 additional diagnoses *)	7	11.5
* Hepatic congestion (1), enterocolitis (1), fungal infection of oral keratin (1), hepatic necrosis (1), hepatitis (1), persistently elevated liver biochemistry markers of unknown aetiology (1).			
**Urinary**			
	*Urinary, combined*	*9*	*14.8*
	Interstitial mononuclear nephritis	4	6.6
	Nephrocalcinosis	3	4.9
	Other (9 additional diagnoses *)	9	14.8
* Acute tubular injury (1), haematuria (1), interstitial congestion of the kidney (1), interstitial mononuclear cell aggregates/accumulations (1), intraluminal pigment accumulation (1), mononuclear cells in connective tissue of bladder (1), renal hyperaemia (1), renal mineralisation (radiographic finding) (1), renal tubular degeneration and necrosis (1).			
**Generalised**			
	*Generalised, combined*	*8*	*13.1*
	Other (8 diagnoses *)	10	16.4
* Emaciation (2), possible torpor (2), chemical irritation (chlorine) (1), died following entrapment in a permanent human-made structure (1), male conspecific trauma (1), poor body condition attributable to harassment by conspecific (1), sepsis (1), trauma: conspecific (1).			
**Musculoskeletal**			
	*Musculoskeletal, combined*	7	*11.5*
	Other (5 diagnoses *)	7	11.5
* Bacterial myositis (2), degenerative joint disease (2), fracture/s (1), myopathy (1), saponification (1).			
**Haemolymphatic**			
	*Haemolymphatic, combined*	*6*	*9.8*
	Splenic histiocytosis	3	4.9
	Other (5 additional diagnoses *)	6	9.8
* Splenic lymphoid hyperplasia (2), splenic fibrin deposits (1), splenic lymphocytolysis (1), splenic plasma cell hyperplasia (1), splenic round cell tumour (1).			
**Respiratory**			
	*Respiratory, combined*	*6*	*9.8*
	Pulmonary congestion	3	4.9
	Pulmonary oedema	3	4.9
	Other (3 additional diagnoses *)	3	4.9
* Mediastinal oedema (1), pneumonia—bacterial (1), pulmonary interstitial reaction (1).			
**Endocrine**			
	*Endocrine, combined*	*3*	*4.9*
	Other (5 diagnoses *)	6	9.8
* Adrenal cortical intranuclear inclusion bodies (2), adrenal karyomegaly (1), adrenal medullary necrosis (1), thyroid hyperplasia (1), thyroid haemorrhage (1).			
**Cardiovascular**			
	*Cardiovascular, combined*	*2*	*3.3*
	Other (2 diagnoses *)	2	3.3
* Arteriosclerosis (1), myocarditis (1).			
**Nervous**			
	*Nervous, combined*	*2*	*3.3*
	Other (2 diagnoses *)	2	3.3
* Meningoencephalitis (1), neuronal lipofuscinosis (1).			
**Sensory**			
	*Sensory, combined*	*2*	*3.3*
	Other (2 diagnoses *)	2	3.3
* Cataracts (1), conjunctivitis (1).			
**Reproductive**			
	*Respiratory, combined*	*1*	*1.6*
	Other (1 diagnosis *)	1	1.6
* Unfertilised eggs (1).			

**Table 12:** The aetiological category and subcategory for diagnoses in zoo-housed platypuses (high diagnostic confidence presentations only), by body system (*n* = 61). Total % calculated as a percentage of the total number of presentations (i.e., 61). Categories are listed in alphabetical order.

		Body System												
Category	Sub-Category	Generalised	Integumentary	Musculoskeletal	Nervous	Cardiovascular	Respiratory	Digestive	Urinary	Endocrine	Sensory	Reproductive	Haemolymphatic	Total by Category
Congenital		0	0	0	0	0	0	0	0	0	0	0	0	0 (0%)
Degenerative		0	0	2	1	1	0	0	0	0	1	0	0	5 (8.2%)
Environmental		0	0	0	0	0	0	0	0	0	0	0	0	0 (0%)
Iatrogenic		0	2	0	0	0	0	0	0	0	0	0	0	2 (3.3%)
Indeterminate		0	7	2	0	0	2	1	0	0	0	0	0	12 (19.7%)
Infectious	Bacterial	1	3	2	0	0	1	1	0	0	0	0	1	9 (14.8%)
Infectious	Viral	0	0	0	0	0	0	0	0	0	0	0	0	0 (0%)
Infectious	Fungal	0	17	0	1	0	0	1	0	0	0	0	0	19 (31.2%)
Infectious	Metazoan parasite	0	0	0	0	0	0	0	0	0	0	0	0	0 (0%)
Infectious	Protozoan parasite	0	0	0	0	0	0	1	0	0	0	0	0	1 (1.6%)
Infectious	Unidentified	0	0	0	0	0	0	0	0	0	0	0	0	0 (0%)
Inflammatory		0	0	0	0	0	0	0	0	0	0	0	0	0 (0%)
Metabolic		1	1	0	0	0	0	0	0	0	0	0	0	2 (3.3%)
Neoplastic		0	1	0	0	0	0	0	0	0	0	0	1	2 (3.3%)
Egg death	Undiagnosed	0	0	0	0	0	0	0	0	0	0	1	0	1 (1.6%)
Nutritional		2	0	0	0	0	0	0	3	0	0	0	0	5 (8.2%)
Reproductive		0	0	0	0	0	0	0	0	0	0	0	0	0 (0%)
Toxic		1	0	0	0	0	0	0	0	0	0	0	0	1 (1.6%)
Trauma	Other/ unknown	0	4	1	0	0	0	0	0	0	0	0	0	5 (8.2%)
Trauma	Entrapment in a permanent, human-made structure	1	0	0	0	0	0	0	0	0	0	0	0	1 (1.6%)
Trauma	Self or conspecific	3	8	0	0	0	0	1	0	0	0	0	0	12 (19.7%)
Trauma	Vehicular	0	0	0	0	0	0	0	0	0	0	0	0	0 (0%)
Undetermined		0	21	0	0	1	0	4	10	2	1	0	0	39 (63.9%)
Total by body system		9 (14.8%)	43 (70.5%)	7 (11.5%)	2 (3.3%)	2 (3.3%)	3 (4.9%)	9 (14.8%)	13 (21.3%)	2 (3.3%)	2 (3.3%)	1 (1.6%)	2 (3.3%)	

## Data Availability

Restrictions apply to the availability of the data as records were provided by multiple institutions and individuals. Requests to access the datasets should be directed to the corresponding author.

## Abbreviations

The following abbreviations are used in this manuscript:

ACT	Australian Capital Territory
IUCN	International Union for Conservation of Nature
NSW	New South Wales
TVI	Tail Volume Index
WHA	Wildlife Health Australia
ZIMS	Zoological Information Management System

## References

[1] Bino G., Kingsford R.T., Archer M., Connolly J.H., Day J., Dias K., Goldney D., Gongora J., Grant T., Griffiths J. (2019). The Platypus: Evolutionary History, Biology, and an Uncertain Future. J. Mammal..

[2] Woinarski J., Burbidge A.A. *Ornithorhynchus anatinus*. The IUCN Red List of Threatened Species 2016. https://www.iucnredlist.org/species/40488/21964009.

[3] Jackson S., Thomas J.L., Serena M., Temple-Smith P. (2025). Platypus. Australian Mammals: Biology and Captive Management.

[4] Grant T., Serena M., Williams G., Temple-Smith P. (2024). Age Determination in the Platypus (*Ornithorhynchus anatinus*) Using Spur Sheath and Spur Developmental Stages: A Review. Aust. Mammal..

[5] Whinfield J., Warren K., Vogelnest L., Vaughan-Higgins R. (2024). Applying a Modified Streamlined Disease Risk Analysis Framework to a Platypus Conservation Translocation, with Special Consideration for the Conservation of Ecto- and Endoparasites. Int. J. Parasitol. Parasites Wildl..

[6] Ryser-Degiorgis M.-P. (2013). Wildlife Health Investigations: Needs, Challenges and Recommendations. BMC Vet. Res..

[7] Kessell A.E., Boulton J.G., Dutton G.J., Woodgate R., Shamsi S., Peters A., Connolly J.H. (2014). Haemolytic Anaemia Associated with *Theileria* sp. in an Orphaned Platypus. Aust. Vet. J..

[8] McColl K.A. (1983). Pathology in Captive Platypus (*Ornithorhynchus anatinus*) in Victoria, Australia. J. Wildl. Dis..

[9] Connolly J., Obendorf D., Whittington R., Muir D. (1997). Causes of Morbidity and Mortality in Platypus (*Ornithorhynchus anatinus*) from Tasmania, with Particular Reference to *Mucor amphibiorum* Infection. Aust. Mammal..

[10] Paparini A., Macgregor J., Irwin P.J., Warren K., Ryan U.M. (2014). Novel Genotypes of *Trypanosoma binneyi* from Wild Platypuses (*Ornithorhynchus anatinus*) and Identification of a Leech as a Potential Vector. Exp. Parasitol..

[11] Macgregor J.W., Holyoake C.S., Munks S.A., Connolly J.H., Robertson I.D., Fleming P.A., Warren K.S. (2017). Investigation into Individual Health and Exposure to Infectious Agents of Platypuses (*Ornithorhynchus anatinus*) in Two River Catchments in Northwest Tasmania. J. Wildl. Dis..

[12] Whittington R.J., Grant T.R., McKercher J., Suann M., Hart K., Handasyde K.A., Macgregor J., Westman M.E., Connolly J.H. (2024). Leptospirosis in the Platypus (*Ornithorhynchus anatinus*) in Australia: Who Is Infecting Whom?. Animals.

[13] Gust N., Griffiths J., Driessen M., Philips A., Stewart N., Geraghty D. (2009). Distribution, Prevalence and Persistence of Mucormycosis in Tasmanian Platypuses (*Ornithorhynchus anatinus*). Aust. J. Zool..

[14] Scheelings T.F. (2016). Morbidity and Mortality of Monotremes Admitted to the Australian Wildlife Health Centre, Healesville Sanctuary, Australia, 2000–2014. Aust. Vet. J..

[15] Serena M., Williams G. (2010). Factors Contributing to Platypus Mortality in Victoria. Victorian Nat..

[16] Serena M., Williams G.A., Thomas J.L. (2025). Factors Contributing Directly to Platypus (*Ornithorhynchus anatinus*) Mortality and Implications for Conserving Populations in the Wild. Aust. Mammal..

[17] Vitali S., Reiss A., Jakob-Hoff R., Stephenson T., Holz P., Higgins D. (2023). National Koala Disease Risk Analysis Report Version 1.2.

[18] Gearing R., Mian I., Barber J., Ickowicz A. (2006). A Methodology for Conducting Retrospective Chart Review Research in Child and Adolescent Psychiatry. J. Can. Acad. Child Adolesc. Psychiatry.

[19] Talari K., Goyal M. (2020). Retrospective Studies—Utility and Caveats. J. R. Coll. Physicians Edinb..

[20] Griffith J.E., Dhand N.K., Krockenberger M.B., Higgins D.P. (2013). A Retrospective Study of Admission Trends of Koalas to a Rehabilitation Facility Over 30 Years. J. Wildl. Dis..

[21] Gilbert E.H., Lowenstein S.R., Koziol-McLain J., Barta D.C., Steiner J. (1996). Chart Reviews in Emergency Medicine Research: Where Are The Methods?. Ann. Emerg. Med..

[22] McCreesh K., Guthrie A.L., Spiro S., Patterson S. (2024). A Systematic Review of Retrospective Morbidity and Mortality Studies on Captive Wildlife Species. J. Zoo Wildl. Med..

[23] Vandenbroucke J.P., von Elm E., Altman D.G., Gøtzsche P.C., Mulrow C.D., Pocock S.J., Poole C., Schlesselman J.J., Egger M., Initiative S. (2007). Strengthening the Reporting of Observational Studies in Epidemiology (STROBE): Explanation and Elaboration. PLoS Med..

[24] ZIMS Species Holdings Species360 Zoological Information Management System. https://zims.species360.org/.

[25] Wildlife Health Australia eWHIS—Wildlife Health Information System. https://www.wildlifehealthaustralia.com.au/ProgramsProjects/eWHIS-WildlifeHealthInformationSystem.aspx#requests.

[26] Booth R., Connolly J., Vogelnest L., Woods R. (2008). Platypus. Medicine of Australian Mammals.

[27] Vogelnest L., Allan G. (2015). Platypus. Radiology of Australian Mammals.

[28] Macgregor J.W., Holyoake C., Munks S., Connolly J.H., Robertson I.D., Fleming P.A., Lonsdale R.A., Warren K. (2017). Assessing Body Condition in the Platypus (*Ornithorhynchus anatinus*): A Comparison of New and Old Methods. Aust. J. Zool..

[29] Grant T.R., Temple-Smith P.D. (1998). Growth of Nestling and Juvenile Platypuses (*Ornithorhynchus anatinus*). Aust. Mammal..

[30] Thomas J. (2018). Breeding Biology of the Platypus (*Ornithorhynchus anatinus*). Ph.D. Thesis.

[31] Furlan E., Griffiths J., Gust N., Armistead R., Mitrovski P., Handasyde K.A., Serena M., Hoffmann A.A., Weeks A. (2011). Is Body Size Variation in the Platypus (*Ornithorhynchus anatinus*) Associated with Environmental Variables?. Aust. J. Zool..

[32] Thomas J.L., Parrott M.L., Handasyde K.A., Temple-Smith P. (2019). Burrow Use by Juvenile Platypuses (*Ornithorhynchus anatinus*) in Their Natal Home Range. J. Mammal..

[33] Thomas J.L. (2025). Translocation of a Rehabilitated Juvenile Platypus (*Ornithorhynchus anatinus*). Aust. Mammal..

[34] Bethge P., Munks S., Otley H., Nicol S.C. (2004). Platypus Burrow Temperatures at a Subalpine Tasmanian Lake. Proc. Linn. Soc. N.S.W..

[35] Vogelnest L., Vogelnest L., Portas T. (2019). Platypus. Current Therapy in Medicine of Australian Mammals.

[36] Serena M., Williams G.A. (2012). Effect of Sex and Age on Temporal Variation in the Frequency and Direction of Platypus (*Ornithorhynchus anatinus*) Captures in Fyke Nets. Aust. Mammal..

[37] Bino G., Grant T.R., Kingsford R.T. (2015). Life History and Dynamics of a Platypus (*Ornithorhynchus anatinus*) Population: Four Decades of Mark-Recapture Surveys. Sci. Rep..

[38] Vogelnest L., Whinfield J., Vogelnest L., Portas T. (2025). Platypus. Current Therapy in Medicine of Australian Mammals: Revised Edition.

[39] Saverimuttu S., McInnes K., Warren K., Yeap L., Hunter S., Gartrell B., Pas A., Chatterton J., Jackson B. (2025). Enabling near Real Time Use of Wildlife Necropsy Data: Text-Mining Approaches to Derive Interactive Dashboard Displays. PLoS ONE.

